# Orthotopic Heart Transplantation in a Patient With Gitelman Syndrome and Dilated Cardiomyopathy

**DOI:** 10.1177/2150135120912227

**Published:** 2020-07-07

**Authors:** Shannon Alexandra Solt, Timothy M. Hoffman, Mahesh S. Sharma, Katherine D. Westreich, Margaret Kihlstrom, Stephanie P. Schwartz

**Affiliations:** 1 University of North Carolina Medical Center, Chapel Hill, NC, USA

## Abstract

Gitelman syndrome (GS) is a rare hereditary tubulopathy affecting the distal tubule leading to significant electrolyte disturbances.^1^ Although generally a benign condition, rare associations with arrhythmias and sudden cardiac death have been reported.^1^ A paucity of literature exists associating GS with cardiomyopathy. We present a child with dilated cardiomyopathy and GS who was successfully treated with orthotopic heart transplantation.

## Case Report

**Figure 1.**
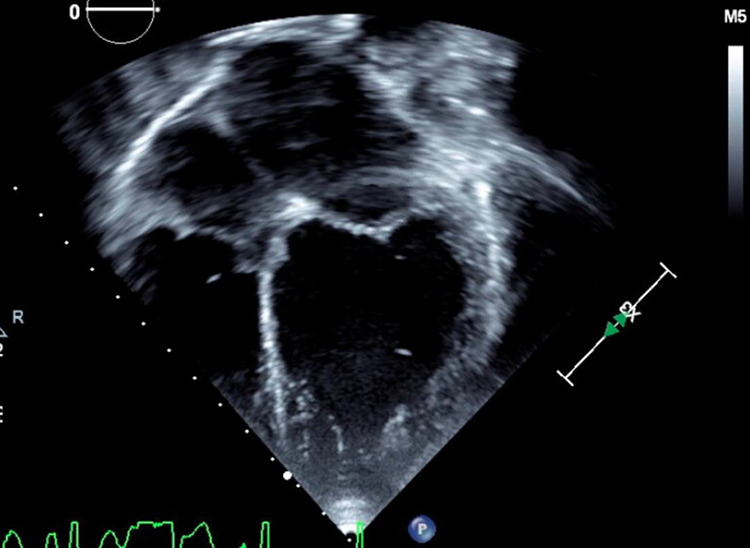
Echocardiogram of patient with dilated cardiomyopathy.

A seven-year-old male with Gitelman syndrome (GS) presented with acute onset right-sided hemiplegia and aphasia. Head computed tomography revealed an ischemic stroke of the left middle cerebral artery. Echocardiogram demonstrated dilated cardiomyopathy, severely depressed biventricular function (ejection fraction: 18%), and no atrial shunt (Figure 1). Echocardiogram two years prior revealed normal biventricular function. Extensive evaluation for an underlying etiology including viral studies and a comprehensive cardiomyopathy sequencing panel were unremarkable.

He was stabilized on a milrinone infusion, then transitioned to enteral heart failure medications and magnesium supplementation, as well as prophylactic anticoagulation for potential cardioembolic stroke. At discharge, he had mild expressive aphasia and right-sided weakness. Within four months, he experienced decompensated heart failure requiring readmission for transplant evaluation and listing. He was bridged to transplant with milrinone and dobutamine infusions, as well as a high-dose magnesium infusion, and listed status 1A by exemption. Due to his history of stroke, a ventricular assist device was not offered as the risk of systemic anticoagulation was felt to outweigh the benefit of mechanical circulatory support.

He was transplanted after seven months of waitlist time. Other than the requirement of multiple magnesium and potassium replacements to maintain serum levels, there were no additional anesthetic or operative management issues related to his GS. Subsequent echocardiograms revealed normal graft function. Posttransplant, the use of tacrolimus in the setting of his underlying GS elicited recalcitrant hypomagnesemia. Postoperative course was complicated by ventricular tachycardia without evidence of rejection, reperfusion injury, or coronary artery abnormality. A lidocaine infusion was initiated and subsequently transitioned to enteral amiodarone. Additionally, he developed significant cramping and tetany in his extremities limiting mobility, which improved with higher serum magnesium levels. A magnesium infusion treated both unique postoperative symptoms. Transition to enteral magnesium was challenging with high stool output and resultant metabolic acidosis. Ultimately, he was discharged home 79 days posttransplant (289 hospitalization days including waitlist time) on a regimen of magnesium oxide, magnesium chloride, sodium bicarbonate, and spironolactone, in addition to amiodarone, enalapril, and immunosuppression with steroids, tacrolimus, and mycophenolate mofetil. His neurologic examination continued to improve throughout hospitalization, and at discharge, he had only mild right-sided weakness.

## Discussion

Gitelman syndrome is an autosomal recessive tubulopathy arising from mutations in the *SLC12A3* gene encoding the thiazide-sensitive NaCl cotransporter.^1,2^ The syndrome is characterized by metabolic alkalosis, hypokalemia, hypomagnesemia, and reduced urinary calcium excretion.^1^ While often benign, rare associations with arrhythmias and sudden cardiac death have been reported.^3,4^ No known association between GS and cardiomyopathy exists. This case complements two previously published reports describing children with GS or suspected GS with dilated cardiomyopathies requiring heart transplantation.^5,6^ While etiologies for these cases were described as probable viral origin or idiopathic, each additional case reinforces a possible association between GS and dilated cardiomyopathy.

Cardiac workup for patients with GS is not well established. Electrocardiograms are suggested for patients with GS and hypomagnesemia to assess QT interval and subsequent arrhythmia risk.^3^ No standard exists for echocardiography. More data are needed to evaluate whether periodic screening echocardiography is indicated.

Many patients with GS remain hypomagnesemic despite supplementation, often requiring large quantities of magnesium in order to reach the lower limit of normal.^1,5^ Renal magnesium wasting and high stool output due to enteral magnesium supplementation often exacerbate hypomagnesemia, leading to the consideration of potassium-sparing diuretics and nonsteroidal anti-inflammatory drugs to curtail renal potassium and magnesium loss.^7,8^ Our patient’s posttransplant hypomagnesemia was exacerbated by the use of a calcineurin inhibitor, a drug class known to induce urinary magnesium wasting.^9^

In the setting of arrhythmias and/or cardiomyopathy, electrolyte balance is critical.^3^ Hypomagnesemia can cause prolongation of the ventricular action potential and subsequent QT interval prolongation.^4^ QTc prolongation is associated with severe ventricular arrhythmias, for which patients posttransplant are already at risk.^4^ Hypomagnesemia is known to cause tetany, cramps, paresthesias, and muscle pain,^1,5^ which can complicate postoperative recovery and patient mobilization.

Our patient’s postoperative ventricular tachyarrhythmias as well as pain and cramping in his extremities were presumed to be exacerbated by hypomagnesemia. Therefore, we initially sought to keep his serum magnesium levels above 2.0 mEq/L, requiring a magnesium sulfate infusion of up to 13.5 mg/kg/h. This level was unachievable with oral supplementation, as increasing doses of enteral magnesium (up to 33 mg/kg/d magnesium chloride and 73 mg/kg/d magnesium oxide) led to intolerable stooling patterns which threatened volume and acid–base status. He was discharged achieving goal magnesium levels of 1.2 mEq/L with six to seven loose stools daily. Subsequently, his serum magnesium level has improved to average 1.7 mEq/L, and the amount of magnesium supplementation required has dramatically decreased.

Salt wasting, an additional characteristic of GS, has the potential to effect volume status.^2^ Liberal sodium intake is encouraged. Renin–angiotensin–aldosterone system inhibitors can aggravate renal sodium wasting and increase the risk of symptomatic hypovolemia but limit renal potassium losses in the distal tubules.^2^ While not first-line treatment for tubulopathies, they are not contraindicated pending hemodynamic tolerance from a hemodynamic standpoint. In our patient, enalapril was started for afterload reduction posttransplant and was well tolerated.

This report provides an additional case of a patient with GS presenting with dilated cardiomyopathy, ultimately receiving a heart transplantation. It is possible that while previously unrecognized, an association exists between GS and dilated cardiomyopathy. Furthermore, while the hypomagnesemia induced by GS and calcineurin inhibitors is challenging to manage postoperatively, heart transplantation is a viable therapeutic option for this patient population.
